# The Assessment and Management of Simple Elbow Dislocations

**DOI:** 10.2174/1874325001711011373

**Published:** 2017-11-30

**Authors:** Andrew J Grazette, Alex Aquilina

**Affiliations:** University Hospitals Coventry and Warwickshire, Clifford Bridge Road, CV2 2DX, Coventry, UK

**Keywords:** Simple Dislocation, Elbow joint, Elbow Dislocations, Epidemiology, Fracture, Surgical intervention

## Abstract

**Background:**

Simple elbow dislocations are a commonly seen joint dislocation involving a sequential disruption of the soft tissue stabilisers without a significant associated fracture.

**Methods:**

A selective literature search was performed and personal surgical experiences are reported.

**Results:**

The majority of these injuries can be treated with expedient closed reduction, with the intact bony congruency of the elbow joint conferring early stability. Early mobilisation after reduction results in a faster recovery with good functional outcomes. Surgical intervention for persistent instability or stiffness is uncommonly required. Although, early surgical ligamentous repair has been considered, the current evidence does not demonstrate any long-term benefits compared to non-operative treatment.

**Conclusion:**

The majority of simple elbow dislocations can be successfully managed non-operatively with good reliable outcomes. Careful follow up is essential, however, to identify patients that may occasionally develop persistent instability or stiffness and require intervention.

## 1 INTRODUCTION

### 1.1 Epidemiology and Classification

Elbow dislocations are uncommon injuries with the annual incidence being 6.1 per 100,000 per annum across all ages [1]. They are however the most commonly dislocated joint in children and the second commonest in adults, after the shoulder. There is predominance of elbow dislocations in males, being 2 - 2.5 times more common in men, and also during the younger years with a mean age of 30. They are usually due to a fall onto the outstretched arm and around 40% are as a result of sporting injury [2, 3].

The elbow joint, despite its potential for dislocation, is inherently stable due to the ulno-humeral joint congruency, the medial collateral ligament (MCL) and the lateral collateral ligament (LCL) complex (including the lateral ulna collateral ligament (LUCL)) – the primary stabilisers. These are complemented by the secondary stabilisers; the radial head, the joint capsule and the common flexor and extensor origins. This is also augmented by dynamic stability provided by the muscles crossing the elbow joint.

Elbow dislocations can be categorised as either simple or complex. Simple elbow dislocations are typically described by the absence of a major associated fracture. Minor bony injuries do sometimes occur in conjunction with simple dislocations and have been described in up to 10% of cases. These usually comprise avulsion fractures from either the medial or lateral epicondyle or the coronoid tip. These fractures are usually insignificant [4]. Given this fact, an alternative description used is that a dislocation is simple if the elbow is stable throughout a full range of movement after relocation [5]. More complex injuries with significant fractures of the radial head or neck, olecranon, coronoid, humeral condyles or epicondyles occur in around 20% of cases [6]. Simple or complex dislocations may also cause damage to the articular surface or small osteochondral injuries imperceptible on plain radiographs. The significance of such injuries is unknown [7].

Elbow dislocations can be further described by the direction of displacement of the radius and ulna relative to the humerus. Posterior dislocations are by far the most common and can be further sub-divided into posterolateral and posteromedial. Posterolateral accounts for 80% of cases, with the remaining 20% made up of posteromedial, medial, anterior, lateral or divergent [8].

Dislocation usually results from an axial loading force across the elbow joint combined with an external rotation/supination force through the forearm and a valgus stress. The pathoanatomy of elbow dislocation has been described as a progressive disruption of the ring of soft tissue or bone around the elbow in three stages [9]:

**Stage 1** – complete disruption of the LUCL with complete or partial disruption of the remaining LCL, resulting in posterolateral rotatory subluxation of the elbow.
**Stage 2** – additional disruption to the capsule both anteriorly and posteriorly resulting in an incomplete posterolateral dislocation with the coronoid perched on the trochlea.
**Stage 3** – disruption of the MCL resulting in posterior dislocation of the elbow.

## 2 INITIAL ASSESSMENT AND MANAGEMENT

Elbow dislocation may present as an isolated injury or as one of many injuries sustained in the polytrauma patient. Appropriate assessment and management of these patients along trauma algorithms may be necessary. A detailed history of the mechanism of injury is beneficial, and information regarding the patient’s functional status can be helpful in guiding treatment.

**Fig. (1):**
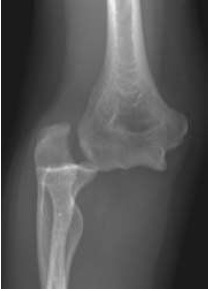
Antero-posterior and lateral radiographs showing postero-lateral simple elbow dislocation.

**Fig. (2):**
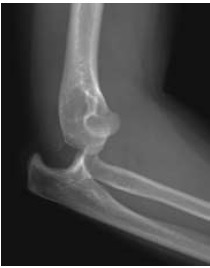
Antero-posterior and lateral radiographs showing postero-lateral simple elbow dislocation.

On clinical examination, the dislocated elbow will be deformed with the forearm typically described in a position of varus and supination for postero-lateral dislocations. Careful assessment and documentation of neurovascular status should be completed prior to, and following, reduction as entrapment of neurovascular structures can occur and necessitates urgent surgical management. Other injuries to the limb should be sought, with particular focus on the distal radio-ulnar joint (DRUJ) to assess for interosseous membrane injury. Radiographs should be used to confirm the extent of the injury, and in simple elbow dislocations anteroposterior and lateral radiographs usually suffice. In more complex injuries computed tomography can often be beneficial in planning further management (Figs. **1** and **2**).

**Fig. (3):**
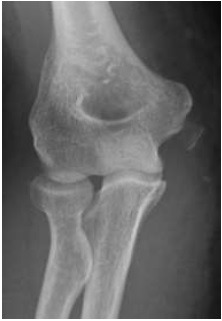
Antero-posterior and lateral radiographs after closed reduction of a simple elbow dislocation – note medial epicondyle avulsion fracture.

**Fig. (4):**
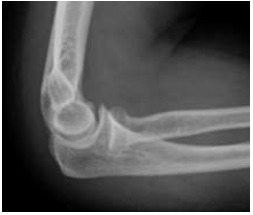
Antero-posterior and lateral radiographs after closed reduction of a simple elbow dislocation – note medial epicondyle avulsion fracture.

**Fig. (5):**
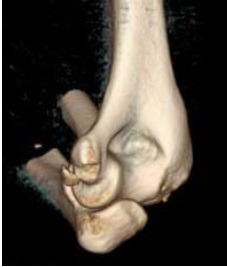
Three-dimensional computed tomography scan showing medial epicondyle avulsion following reduction of a simple elbow dislocation (this has ruled out any significant osseous injuries).

Reduction of the dislocation to stable congruent joint is the aim of treatment. Simple dislocations of the elbow are usually reduced in the emergency department with analgesia and conscious sedation, however, manipulation can be performed under a general anaesthetic in the operating theatre, and this allows a more comprehensive examination under anaesthesia of the reduced joint. In the adequately sedated patient, reduction is achieved by correction of any medial or lateral displacement, flexion of the elbow to approximately 25 degrees, supination of the forearm and longitudinal traction with countertraction of the upper arm by an assistant [3]. A reduction ‘clunk’ may be heard. Clinical and radiographic examination should then be repeated to confirm the reduction (Figs. **3** and **4**). Occasionally, a computed tomography scan may be required to gain more information about any associated fractures and their significance (Fig. **5**).

In simple dislocations, the bony congruency is often sufficient to achieve and maintain a stable closed reduction, however, the elbow should be examined for signs of instability. It should be taken through a gentle full range of motion in the flexion-extension plane. If dislocation reoccurs, the degree of extension causing dislocation should be recorded and then re-examined with the forearm in pronation. If the elbow remains unstable with the forearm in pronation and greater than 45 degrees of elbow flexion, then early surgical intervention with ligamentous repair, reconstruction or external fixation is indicated. Varus and valgus stress testing in extension and 30 degrees of flexion should also be performed as well as posterolateral rotatory stress testing by means of the lateral pivot-shift manoeuvre [3, 8, 10].

Following a stable reduction, the elbow can be rested in a sling, splint or bulky bandage for 3-7 days [11]. If the elbow is unstable between 45 degrees of flexion and full extension then an extension block combined with a hinged brace can be used [8]. Full plasters should be avoided due to the potential to pull the elbow into a subluxated position. Immobilisation of any form for more that 2-3 weeks should also be avoided, as it is associated with a poorer outcome [4, 12, 13]. Indeed, in a recent multicenter randomized control trial comparing early active mobilization to plaster immobilization for 3 weeks, patients in the early active mobilization group demonstrated a significantly greater range of movement, a faster recovery and faster return to work [14].

## 3 OUTCOMES AND COMPLICATIONS

For the majority of patients, functional treatment alone has been shown to provide good outcomes in long-term studies. 50% of patients gain an excellent range of movement (with a loss of less than 5°) and normal strength, and a third of patients have good results (with less than 15° loss of range and minimal discomfort) [11, 12, 15]. The loss of range of movement is typically of the final 10-15 degrees of extension. Despite objectively good outcomes, many patients, however, describe ongoing symptoms, and in a study looking at patient reported outcomes 62% of participants described some residual pain and 56% reported ongoing subjective stiffness at a mean 7 years follow up [5].

Instability is a less common problem than stiffness, but has received more attention in the literature since the anatomical basis of postero-lateral rotatory instability was described [16]. Up to one third of patients may report symptoms of instability [14], but functional instability will depend on the demand on the joint and has been reported at 8% [5]. An even lower proportion of only 2.3% of patients may actually require any form of soft tissue stabilisation procedure for this [17].

Following a stable reduction, early surgical intervention to repair the damaged soft tissues around the elbow has shown no additional benefit over functional treatment whilst conferring the additional risk of an open surgical procedure and should therefore be avoided [18-20]. However, the spectrum of soft tissue injury around elbow dislocation varies widely and there may well be a role of soft tissue repair in the more highly unstable elbow [21]. In cases of soft tissue repair there remains no clear benefit to early surgery in comparison to delayed intervention [22]. The role of ligamentous repair in elbow dislocation would benefit from further study.

A recent population database study of nearly 5000 patients with a minimum 2 year follow up showed that few patients require delayed surgery (3.6%) following a simple elbow dislocation. Only 1.5% of patients had a failed closed reduction requiring subsequent open reduction [23] and 1.2% required contracture releases within 4 years of injury [17].

Other injuries and long-term sequelae do occur and care should be taken in the assessment and follow up of these patients. Neurological injury has been reported in up to 20% of simple dislocations [24]. This is usually a transient paraesthesia of the ulna nerve, but there can be more permanent injury and the median nerve can also be affected. The brachial artery can be damaged due to traction at the time of dislocation or by entrapment following reduction. Emergent complications requiring surgical intervention such as open dislocations or compartment syndrome have been reported, but are very rare. Heterotopic ossification has been documented to occur in up to 75% of cases, but is only motion limiting in around 5% of cases [24] and usually does not require surgical intervention.

## SUMMARY

Simple elbow dislocations are a commonly seen injury within orthopaedic practice and expedient closed reduction and early active mobilisation usually results in excellent or good long-term functional outcomes.

Despite a recent increasing trend for early surgical ligamentous repair, a Cochrane review and further cohort series have shown no benefit to early surgical intervention following stable reduction. Further research is required to fully establish the role and benefits of surgery.

A recent large multi-centre randomized control trial and many cohort studies have shown that early functional treatment is better than immobilization with patient achieving a greater range of movement, faster recovery and a faster return to work. However, patient reported studies suggest that rates of residual pain and subjective stiffness are higher than previously thought.

Assessment for instability must be performed acutely and at follow up, and if present then it should be further investigated. However, the overall number of patients requiring surgical intervention for any cause following simple elbow dislocation is low at 4% with procedures usually performed within 4 years of the injury [17].
